# Genetic Adaptation of a Mevalonate Pathway Deficient Mutant in *Staphylococcus aureus*

**DOI:** 10.3389/fmicb.2018.01539

**Published:** 2018-07-12

**Authors:** Sebastian Reichert, Patrick Ebner, Eve-Julie Bonetti, Arif Luqman, Mulugeta Nega, Jacques Schrenzel, Cathrin Spröer, Boyke Bunk, Jörg Overmann, Peter Sass, Patrice François, Friedrich Götz

**Affiliations:** ^1^Microbial Genetics, Interfaculty Institute of Microbiology and Infection Medicine, University of Tübingen, Tübingen, Germany; ^2^Genomic Research Laboratory, Division of Infectious Diseases, Geneva University Hospital, Geneva, Switzerland; ^3^Leibniz Institute DSMZ-German Collection of Microorganisms and Cell Cultures, Braunschweig, Germany; ^4^Microbial Bioactive Compounds, Interfaculty Institute of Microbiology and Infection Medicine, University of Tübingen, Tübingen, Germany

**Keywords:** adaptation, mutation, mevalonate pathway, isoprenoids, lactonase, Drp35, Spx

## Abstract

In this study we addressed the question how a mevalonate (MVA)-auxotrophic *Staphylococcus aureus*Δ*mvaS* mutant can revert to prototrophy. This mutant couldn’t grow in the absence of MVA. However, after a long lag-phase of 4–6 days the mutant adapted from auxotrophic to prototrophic phenotype. During that time, it acquired two point mutations: One mutation in the coding region of the regulator gene *spx*, which resulted in an amino acid exchange that decreased Spx function. The other mutation in the upstream-element within the core-promoter of the mevalonolactone lactonase gene *drp35*. This mutation led to an increased expression of *drp35*. In repeated experiments the mutations always occurred in *spx* and *drp35* and in the same order. The first detectable mutation appeared in *spx* and allowed slight growth; the second mutation, in *drp35*, increased growth further. Phenotypical characterizations of the mutant showed that small amounts of the lipid-carrier undecaprenol are synthesized, despite the lack of *mvaS*. The growth of the adapted clone, Δ*mvaS*^ad^, indicates that the mutations reawake a rescue bypass. We think that this bypass enters the MVA pathway at the stage of MVA, because blocking the pathway downstream of MVA led to growth arrest of the mutant. In addition, the lactonase Drp35 is able to convert mevalonolactone to MVA. Summarized, we describe here a mutation-based two-step adaptation process that allows resuscitation of growth of the Δ*mvaS* mutant.

## Introduction

The very large and diverse class of isoprenoids is composed of 1000s of different organic compounds. Representatives can be found in all living organisms, where they fulfill essential roles. They are involved in the electron transport during aerobic respiration (quinones), the cell wall synthesis (bactoprenols), the photosynthesis (carotenoids), membrane stabilization, protein translation and degradation, gene transcription and many more (Holstein and Hohl, 2004). All isoprenoids share a common basic precursor, the five-carbon molecule IPP and its isomer DMAPP. There are two alternative routes for the synthesis of IPP: the classical MVA pathway with mevalonic acid as an intermediary product, and the MEP pathway, also known as deoxyxylulose 5-phosphate (DXP) pathway or non-MVAP (Eisenreich et al., 1996; Schwender et al., 1996). In general, green algae and most eubacteria use the MEP pathway (Lange et al., 2000), whereas mammals, yeast, archaea and some Gram-positive cocci synthesize IPP via the MVA pathway (Boucher and Doolittle, 2000; Wilding et al., 2000). Some organisms such as plants and a few bacterial species contain both pathways.

Staphylococci either use the one or the other pathway dependent on their preferred habitat. *Staphylococcus* species that use the MVA pathway are often associated with humans and primates, whereas the MEP pathway is predominantly found in species linked to companion animals, livestock, and wildlife (Misic et al., 2016). Recently, *Staphylococcus sciuri* strain ATCC 29059 was found to possess the complete sets of genes for both pathways (Christo-Foroux et al., 2017). Both pathways are also present in *Listeria monocytogenes* and some *Streptomycetes* (Begley et al., 2004; Heuston et al., 2012).

The MVA pathway, which is used by *Staphylococcus aureus* for IPP biosynthesis, starts with the acetylation of acetoacetyl-CoA to Hydroxy-3-methylglutaryl-CoA (HMG-CoA) catalyzed by the HMG-CoA synthase (MvaS) (Ferguson et al., 1959). HMG-CoA is then reduced by the HMG-CoA reductase (MvaA) to MVA (Durr and Rudney, 1960). This part is termed the upper MVA pathway. The lower part of the MVA pathway consists of the double phosphorylation of MVA to mevalonate-5-pyrophosphate by two kinases (MvaK1 and MvaK2) (Tchen, 1958; Tada and Lynen, 1961) and the final decarboxylation and dehydration to IPP and DMAPP by the mevalonate decarboxylase (MvaD) (Bloch et al., 1959). The reaction cascade is shown in Supplementary Figure S1A for better understanding.

Because of the importance of isoprenoids during different cellular processes the biosynthesis of IPP is essential for all living organisms.

In *S. aureus*, for example, repressing the expression of each of the MVA pathway genes drastically reduced growth, and a temperature-sensitive *mvaA* mutant was unable to grow at high temperatures (Balibar et al., 2009; Matsumoto et al., 2016).

In an earlier study we created a deletion mutant (*ΔmvaS*) in *S. aureus* and could show that this mutant was unable to grow in the absence of MVA in the medium, meaning that the mutant was auxotrophic for MVA (Yu et al., 2013). Surprisingly, after prolonged cultivation we obtained stable *ΔmvaS* variants that were able to grow without MVA, which suggests unknown mechanisms for compensating undecaprenol synthesis without MVA in *S. aureus*. This work demonstrated how flexible and adaptable bacteria are in order to survive under nutrient depletion.

Now, we investigated the adaptation process during the prolonged lag-phase in which the MVA auxotrophic Δ*mvaS* becomes prototrophic. We hypothesized that the adaptation is the result of mutations or gene amplifications, because long-term adaptation of bacteria to a certain environment is often based on such genetic variations (Wray, 2007; Andersson and Hughes, 2009). We could show that two sequential point mutations occurred in the regulator gene *spx* and the MVA lactonase *drp35*. Although the adapted mutant, Δ*mvaS*^ad^, became prototrophic, its phenotypic characterization implies a weakened cell wall biosynthesis and increased osmotic stress susceptibility.

## Materials and Methods

### Bacterial Strains, Plasmids and Growth Conditions

All the bacterial strains used in this study are listed in Supplementary Table S1. *S. aureus* HG001 (Herbert et al., 2010) was used as parent strain. *Escherichia coli* DC10B (Monk et al., 2012) was used as a cloning host for the shuttle vectors pBASE6 (Geiger et al., 2012), pPTtuf (Popella et al., 2016) and pRAB11 (Helle et al., 2011). Their derivatives were constructed using Gibson assembly (Gibson et al., 2009). *E. coli* cells were grown at 37°C in basic medium [BM, 1% (w/v) casein peptone, 0.5% (w/v) yeast extract, 0.5% (w/v) NaCl, 0.1% (w/v) K_2_HPO_4_, 0.1% (w/v) glucose, pH 7.2], *S. aureus* cells were grown at 37°C in tryptic soy broth (TSB, Fluka) or BM. *E. coli* cultures were supplemented with ampicillin (100 μg/ml), when appropriate. In case of *S. aureus* chloramphenicol (10 μg/ml, pBASE6, pRAB11) or tetracycline (25 μg/ml, pPTtuf) was added.

### Construction of Plasmids, Knock-Out and Knock-In Mutants

Oligonucleotides and plasmids are listed in Supplementary Tables S1, S2. The construction of the knock-in (*drp35^c-41t^*, *spx^T11I^* and *drp35^c-41t^*/*spx^T11I^*) and knockout mutants (Δ*spx*, Δ*mvaS*^ad^Δ*mvaA*) in HG001 and Δ*mvaS* was performed using the plasmid pBASE6 and allelic replacement as described in (Bae and Schneewind, 2006). Briefly, around 1 kb of the upstream and downstream regions were amplified and ligated into the *BglII*-site of pBASE6. As template, chromosomal DNA of HG001 or *ΔmvaS*^ad^ was used. The knock-in plasmids were constructed by amplifying the mutated site together with roughly 1 kb of each flanking region and ligating it into the *BglII*-site of pBASE6. Over-expression plasmids were constructed by amplifying the desired gene together with a Shine-Dalgarno sequence (AGGAGGT) and ligating it into the *BglII*-site of pRAB11 or the *EcoRI*-site of pPTtuf. The resulting plasmids pBASE_*drp35*-KO, pBASE_*spx*-KO, pBASE_*mvaA*-KO, pBASE_*drp35^c-41t^*-KI, pBASE_*spx^T11I^*-KI, pPTtuf_*drp35*-strep and pRAB11_*spx*^DD^ were transformed into the appropriate strains by electroporation.

### Selection of Adapted *ΔmvaS* Mutants

Adapted *mvaS* mutants (*ΔmvaS*^ad^) were selected as described before (Yu et al., 2013) with some modifications. Briefly, *S. aureus ΔmvaS* was incubated in TSB, supplemented with 500 μM (±)-MVL (Sigma–Aldrich), and grown overnight. Afterward, *ΔmvaS* was inoculated into fresh TSB without MVL to an OD_578_ of 0.005 to keep the influence of residual MVL as low as possible. This culture was incubated for several days at 37°C under aerobic conditions and the OD_578_ was monitored every 24 h. As soon as the culture reached the stationary growth phase a fresh culture was inoculated, and it was streaked on tryptic soy agar (TSA) plates to get single colonies. After several days of incubation one of the biggest colonies was taken, streaked again and used for all upcoming experiments.

### Growth Studies

Growth experiments with the *ΔmvaS* knock-in mutants were done by incubating the cells in TSB, supplemented with 500 μM (±)-MVL (TSB_MV A_), overnight. Cultures were then diluted to an OD_578_ of 0.01 and further serially diluted 1:10. Finally, 10 μl of each dilution was dropped on a TSA plate and incubated at 37°C for 5 days.

### Super-Resolution Fluorescence Microscopy

Cells were grown to the mid-exponential growth phase and washed with PBS. The pellets were then resuspended in PBS and incubated with BODIPY^TM^ FL Vancomycin (0.25 μg/ml, Invitrogen) for cell wall staining and FM5-95 (7 μg/ml, Molecular Probes) for membrane staining for 10 min at 37°C. To remove unbound dye cells were washed twice in PBS and finally resuspended in PBS. For fluorescence microscopy, bacteria were mounted on microscope slides covered with a thin film of 2% agarose dissolved in PBS. Fluorescence micrographs were obtained using a Zeiss Axio Observer Z1 LSM 800 equipped with Airyscan detector and C Plan-Apo 63x/1.4 Oil DIC objective (Zeiss). Image acquisition and analysis were performed via ZEN 2.3 image analysis software package (Zeiss).

### RNA-Isolation for Microarray

RNA for microarray was isolated from bacterial cultures of HG001 and its Δ*mvaS*^ad^ mutant during mid- and late exponential growth phase using the acid guanidinium thiocyanate-phenol-chloroform extraction method described in Schuster and Bertram (2014). DNA was removed by DNaseI digestion as described in Fischer et al. (2011). Pools of 5 μg total RNA for each condition were reverse-transcribed using SuperScript II (Invitrogen, Basel, Switzerland).

### Microarray Manufacturing and Microarray Design

The microarray was manufactured by *in situ* synthesis of 60-base-long oligonucleotide probes (Agilent, Palo Alto, CA, United States), selected as previously described (Charbonnier et al., 2005). The microarray consists in a 15′600 glass slide covering > 95% of all open reading frames (ORFs) annotated in strains NCTC8325, UAMS-1 and SA564 as well as Newman, including their respective plasmids.

### Preparation of Labeled Nucleic Acids for Expression Microarrays

Total RNA was purified from the strains HG001 and Δ*mvaS*^ad^ from two independent cultures. After additional DNase treatment, the absence of remaining DNA traces was confirmed by quantitative PCR with an assay specific for 16S rRNA (Sassi et al., 2015). Batches of 5 μg of total *S. aureus* RNA were labeled by Cy3-dCTP using SuperScript II (Invitrogen, Basel, Switzerland) following the manufacturer’s instructions. Labeled products were then purified onto QiaQuick columns (Qiagen). Purified genomic DNA from the different sequenced strains used for the design of the microarray was extracted (DNeasy; Qiagen), labeled with Cy5 dCTP using the Klenow fragment of DNA polymerase I (BioPrime, Invitrogen, Carlsbad, CA, United States), and used for the normalization process (Talaat et al., 2002). Cy5-labeled DNA (500 ng) and a Cy3-labeled cDNA mixture were diluted in 50 μl of Agilent hybridization buffer and hybridized at a temperature of 60°C for 17 h in a dedicated hybridization oven (Robbins Scientific, Sunnyvale, CA, United States). Slides were washed, dried under nitrogen flow, and scanned (Agilent, Palo Alto, CA, United States) using 100% photon multiplier tube power for both wavelengths.

### Microarray Analysis

Fluorescence intensities were extracted using Feature Extraction software (version 9; Agilent). Local background-subtracted signals were corrected for unequal dye incorporation or unequal load of the labeled product. The algorithm consisted of a rank consistency filter and a curve fit using the default LOWESS (locally weighted linear regression) method. Data consisting of three independent biological experiments were expressed as log 10 ratios and analyzed using GeneSpring, version 8.0 (Silicon Genetics, Redwood City, CA, United States). A filter was applied to select oligonucleotides mapping ORFs in the HG001 genome, yielding approximately 95% coverage. Statistical significance of differentially expressed genes was calculated by analysis of variance (Pohl et al., 2009) using GeneSpring, including the Benjamini and Hochberg false discovery rate correction of 5% (*p*-value cutoff, 0.05) and an arbitrary cutoff of twofold for expression ratios.

### Microarray Data Accession Number

The complete microarray data set has been posted on the Gene Expression Omnibus database^1^ under accession number GSEXXXX for the platform design and GPL10537 for the original data set.

### Reverse Transcription-PCR

Reverse Transcription-PCR (RT-PCR) experiments were carried out using the One*Taq* One-step RT-PCR kit (New England BioLabs, Frankfurt am Main, Germany). Fifty nanogram of RNA was used as template for each reaction. As positive control the housekeeping gene *pykA* was amplified and the One*Taq* DNA Polymerase was taken for the no-RT negative control. Primers used for RT-PCR are listed in Supplementary Table S2. Gene expression was quantified by measuring the strength of the agarose gel bands after incubation in ethidium bromide using ImageJ software.

### Purification and Analysis of Peptidoglycan

Peptidoglycan was isolated from wild type HG001 and the adapted mutant Δ*mvaS*^ad^ as described earlier (de Jonge et al., 1992). Briefly, cells grown up to mid-exponential growth phase were harvested by centrifugation, boiled with 5% SDS for 30 min and broken with glass beads. Broken cells were washed SDS free and resuspended in 100 mM Tris-HCl (pH 7.2) containing 20 mM MgCl_2_ and treated with 10 μg/ml DNAse and 50 μg/ml RNAse for 2 h and subsequently with 100 μg/ml trypsin, 37°C overnight. To remove wall teichoic acid, the PG preparations were incubated with 48% hydrofluoric acid (HFA) for 24 h at 4°C while stirred after washing with water. PG was harvested by centrifugation and washed several times with water until HFA was completely removed and lyophilized. Lyophilized PG was resuspended in 25 mM sodium phosphate buffer (pH 6.8) to a final OD_578_ of 5.0, digested with mutanolysin overnight at 37°C, reduced with sodium borohydride and analyzed by HPLC as described earlier (Nega et al., 2015).

### Extraction, Analysis and Quantification of Bactoprenol

C_55_-isoprenoids were extracted with methanol/chloroform/PBS as described in (Barreteau et al., 2009). Cells were harvested during the exponential growth phase and KOH was used to convert undecaprenyl-pyrophosphate (C_55_-PP) to undecaprenyl-phosphate (C_55_-P) (Kato et al., 1999). The analysis of the C_55_-isoprenoids was performed by reverse-phase HPLC as described (Barreteau et al., 2009) with one major difference. A gradient from 95% buffer A (95% methanol, 5% 2-propanol, 10 mM phosphoric acid) to 100% buffer B (70% methanol, 30% 2-propanol, 10 mM phosphoric acid) in 50 min instead of an isocratic run was developed. The flow rate as well as the column temperature were kept constant at 0.6 ml/min and 30°C, respectively. Undecaprenyl-phosphate was detected at 210 nm. A calibration curve with different amounts of commercial Undecaprenyl-MPDA (monophosphate diammonium) (Larodan, Sweden), which were treated in the same way as the samples, was used to quantify undecaprenol. The amount was projected to nmol of undecaprenol per gram of cell mass dry weight. The KOH-treatment allowed the quantification of C_55_-PP and C_55_-P in one peak.

### Filter Disk Diffusion Assay

To determine the sensitivity of bacterial strains to diamide, an inducer for disulfide stress, and H_2_O_2_ filter disk diffusion assays were performed. Overnight cultures were diluted to an OD_578_ of 0.1 and streaked onto TSA with a cotton swap. Filter disks were prepared by pipetting 20 μl of diamide (50 mM) or H_2_O_2_ (10 mM) onto the disks, which were then put on the agar plates. The diameter of the zone of inhibition (ZOI) were measured after the incubation at 37°C for 18 h using the software ImageJ.

### Purification of Drp35

An overnight culture of *S. aureus* HG001, harboring the plasmid pPTtuf_*drp35*-strep, which constitutively expresses a C-terminal strep-tagged Drp35 protein, was diluted to an OD_578_ of 0.1 with fresh BM and incubated for at least 4 h at 37°C. The cells were harvested by centrifugation (7000 ×*g*, 10 min), washed with phosphate-buffered saline (PBS), and broken down by glass beads using a FastPrep FP120 instrument (MP Biomedicals). To remove cell debris the solution was centrifuged (4°C, 12,000 rpm, 10 min) and the supernatant was subjected to the purification procedure using Strep-Tactin Superflow resin (IBA, Goettingen, Germany) described by the manufacturer. During the whole procedure buffers lacking EDTA were used. After elution the purified protein was dialyzed against 20 mM Tris/HCl, pH 8.0 overnight at 4°C. Protein concentration was determined using the BCA^TM^ Protein Assay Kit (Thermo Scientific).

### Drp35 Activity

Acid production by the hydrolysis of non-aromatic lactones was monitored by a colorimetric assay at 558 nm with correction at 475 nm at 37°C (Draganov et al., 2005). The assay was performed in a Tecan infinite^®^ M200 Microplate Reader, each well contained 2 mM HEPES, pH 8.0, 1 mM CaCl_2_, 0.004% phenol red, 0.005% bovine serum albumin, 1 mM substrate and 5 – 10 μl purified protein. Spontaneous hydrolysis of the substrate was corrected for by substituting the enzyme by the same volume of 20 mM Tris/HCl, pH 8.0. A calibration curve with different amounts of HCl was used to calculate the rate of hydrolysis (Billecke et al., 1999).

The rate of hydrolysis for MVL was also tested for whole protein extracts of different strains. To do so, cells of the early exponential growth phase were harvested and washed twice with 20 mM Tris/HCl, pH 8.0. Afterward, cells were disrupted with glass beads, centrifuged and the supernatant was sterile filtered. Twenty microliter of each cell extract was used for the activity assay.

### *Galleria mellonella* Infection Model

Larvae of *Galleria mellonella* were infected as described previously (Ebner et al., 2016) with few modifications. Bacterial cells of an overnight culture were washed twice with PBS (140 mM NaCl, 10 mM Na_2_HPO_4_, 2.7 mM KCl, 1.8 mM KH_2_PO_4_) and adjusted to a cell concentration of 5 × 10^8^/ml in PBS. The injection volume was 10 μl, which is equal to 5 × 10^6^ cells, per larvae. The larvae were infected with cells of *S. aureus* HG001 and HG001Δ*mvaS*^ad^. In total 30 larvae, separated in three different experiments, were used for each strain. PBS served as a control. After injection, larvae were incubated at 37°C, and dead larvae were counted every 24 h.

### Statistical Analysis

Multiple comparisons were analyzed by either ordinary one-way ANOVA or RM one-way ANOVA with Bonferroni post-test. The log-rank (Mantel-Cox) test was used to analyze the infection model. Statistical analyses were performed with GraphPad Prism software, with significance defined as *p* < 0.05. n represents independent biological replicates.

## Results

### Growth Behavior of the Mevalonate-Prototrophic *S. aureus*Δ*mvaS* Mutant

**FIGURE 1:**
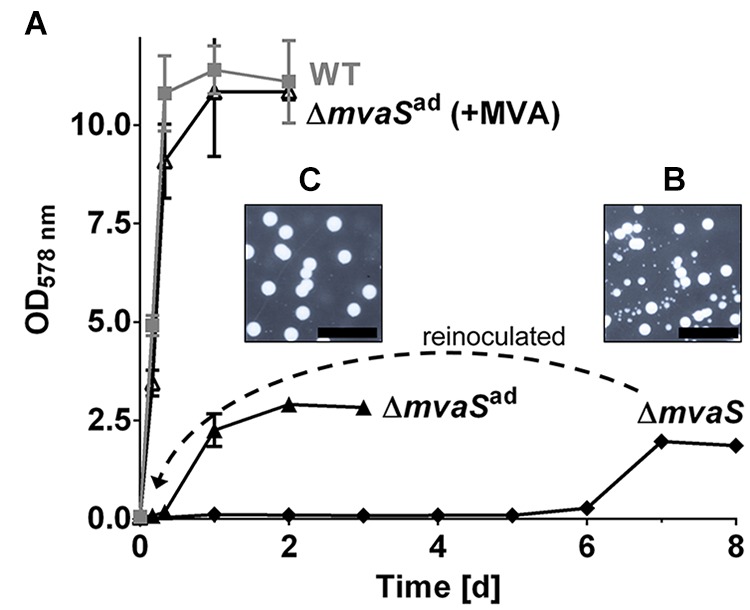
The *mvaS* deletion mutant starts to grow after a prolonged lag-phase of several days. **(A)** Growth curve of HG001, its isogenic *mvaS* deletion mutant and the adapted *mvaS* mutant (*mvaS*^ad^) in the absence and presence of MVA. **(B,C)** Growth on agar plates of Δ*mvaS* **(B)** and Δ*mvaS*^ad^ **(C)** after their cultures reached the stationary phase, incubated for 7 days. For all graphs, except of Δ*mvaS*, each data point is the mean value ± SD; *n* = 3 for WT and Δ*mvaS*^ad^, *n* = 2 for Δ*mvaS*^ad^ (+MVA). The graph of Δ*mvaS* represents one adaptation trial.

Recently we showed that deletion of the *mvaS* gene, encoding the hydroxymethylglutaryl-coenzyme A (HMG-CoA) synthase, leads to auxotrophy for MVA; however, growth of the mutant could be restored by the addition of MVA or MVL to the medium (**Figure 1A**). Surprisingly, after prolonged cultivation for about 4–6 days, the Δ*mvaS* mutant started to grow but reached only an OD_578_ of about 2.5. When samples of this culture were plated on TSA small and large colonies were observed after several days (**Figure 1B**). After inoculation in fresh TSB, the cells were able to grow without a prolonged lag-phase; and when plated on TSA it formed uniformly large colonies (**Figure 1C**). We named these stable variants Δ*mvaS*^ad^, for adapted Δ*mvaS*. Although, Δ*mvaS*^ad^ grew without lag-phase the growth was significantly retarded and they reached only of the OD_578_ of the parent strain (**Figure 1A**). Also, in contrast to the parent strain, colonies of Δ*mvaS*^ad^ were white suggesting that they were not able to produce the orange staphyloxanthin.

### Whole Genome Analysis Revealed Two Important Point Mutations

We hypothesized that adaptation to MVA-prototrophy in Δ*mvaS*^ad^ is due to mutations, why we performed whole genome analysis.

**FIGURE 2:**
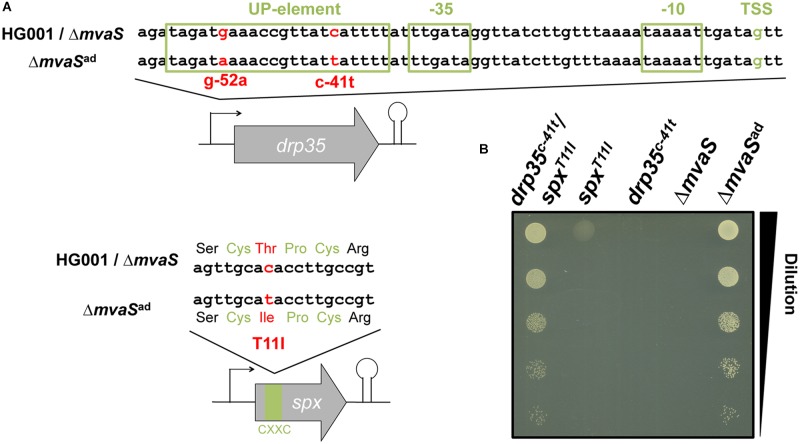
Two point mutations are involved in the adaptation of Δ*mvaS^ad^*. **(A)** Location of the point mutations c-41t in the promoter of the lactonase gene *drp35* and T11I in the C-X-X-C motif of the transcriptional regulator Spx. **(B)** Culture drop test on TS agar plates after back-cloning of *drp35^c-41t^* and *spx^T11I^* separately and together into the non-adapted *mvaS* mutant. Cultures were diluted to an OD_578_ of 0.1, serially diluted 1:10 and dropped on an agar plate, which was incubated aerobically at 37°C.

The genomes of the parent strain HG001, the non-adapted Δ*mvaS* mutant and two independently adapted Δ*mvaS*^ad^ clones from different experiments were sequenced. Isolation of the chromosomal DNA and the sequencing of the genomes were performed by the DSMZ (Braunschweig, Germany) and analyzed for single nucleotide polymorphisms (SNPs). The long-read sequencing technique PacBio RS II with an average read length of 10 kb was used (Rhoads and Au, 2015). For error correction Illumina sequencing was performed. In both Δ*mvaS*^ad^ clones two interesting SNPs could be identified (**Table 1**). One SNP was located in the promoter region of *drp35* (SAOUHSC_03023), which encodes a lactonase-like protein with yet unknown substrate specificity and function. The Δ*mvaS*^ad^ 1-clone carried a base exchange from cytosine to thymine 41 nucleotides upstream of the predicted transcription start site (TSS) (c-41t). The Δ*mvaS*^ad^ 2-clone carried adenine instead of guanine 52 nucleotides upstream of TSS (g-52a) (**Figure 2A** and **Table 1**).

**Table 1:** Mutations found by whole genome sequencing.

Δ*mvaS*^ad^ 1	Δ*mvaS*^ad^ 2
*drp35^c-41t^*	*drp35^g-52a^*
*spx^T11I^*	*spx^T11I^*
*pbp1^N352D^*	*pho^a586g^*


Both Δ*mvaS*^ad^ 1- and 2-clones carried a point mutation in the coding region of *spx* (*SAOUHSC_00934*), which encodes a transcriptional regulator that is involved in the global stress response (Pamp et al., 2006). The SNPs were located at nucleotide position 32 of the coding region where cytosine was exchanged by thymine (c32t). This mutation caused an amino acid exchange in the protein at position 11 from threonine to isoleucine (T11I). This exchange is located within the C-X-X-C motif (amino acids 10–13), which regulates the activity of the protein via the oxidation and reduction of the cysteine residues (Nakano et al., 2005). In Δ*mvaS*^ad^ 1, we additionally found a SNP (N352D) in PBP1 (penicillin binding protein 1) and in Δ*mvaS*^ad^ 2 a silent mutation in *phoR.* Since these two mutations are only present in one mutant they do not contribute to the adaptation and were therefore not considered in further experiments.

### Both Mutations Are Necessary for the Adaptation of Δ*mvaS*^ad^ to MVA-Prototrophy

To confirm the importance of the two mutations in the adaptation of Δ*mvaS*^ad^ to MVA-prototrophy both mutated genes of Δ*mvaS*^ad^ 1, *drp35^c-41t^* and *spx^T11I^*, were introduced separately as well as combined into the non-adapted Δ*mvaS* mutant. The created strains were named Δ*mvaS*-*drp35^c-41t^*, Δ*mvaS*-*spx^T11I^*, and Δ*mvaS*-*drp35^c-41t^/spx^T11I^*. In a serial dilution (1:10) the clones were tested for growth on TSA in the absence of MVA (**Figure 2B**). The Δ*mvaS* clone, which carried both mutations (Δ*mvaS*-*drp35^c-41t^/spx^T11I^*), and Δ*mvaS*^ad^ grew similar. The Δ*mvaS* clone, that carries only *spx^T11I^*, showed only very little growth indicated by the faint spot at the highest cell concentration. The non-adapted Δ*mvaS* mutant as well as Δ*mvaS*-*drp35^c-41t^* were not able to grow. This result indicates that both mutations are necessary for the adaptation of Δ*mvaS*^ad^ to MVA prototrophy.

### Additionally Isolated Δ*mvaS*^ad^ Clones Also Showed Mutations in *spx* and *drp35*

To investigate whether the development of the mutations in *spx* and *drp35* is reproducible we repeated the isolation of adapted Δ*mvaS*^ad^ clones additional five times. In all experiments the adapted clones formed colonies with different size on TSA plates (**Figure 1B**). All tested large colonies revealed SNPs in both genes, *spx* and *drp35*. The identified SNPs were located 40 to 53 nucleotides upstream of the predicted TSS of *drp35*, and in the codons 11 or 14 of *spx*, which always led to an amino acid exchange (**Table 2**).

**Table 2:** Mutations in individually adapted mutants.

	*drp35*		*spx*	

HG001/ Δ*mvaS* (gen.seq.)	-53 tgaaaccgttatca -40		28 tgcacaccttgc 39 (9 SCTPCR 14)	
	Small colony		Large colony	
Mutants	*drp35*	spx	*drp35*	*spx*
Δ*mvaS*^ad^ 1 (gen.seq.)	n.d.	n.d.	c-41t	c32t (T11I)
Δ*mvaS*^ad^ 2 (gen.seq.)	n.d.	n.d.	g-52a	c32t (T11I)
Δ*mvaS*^ad^ 3	–	c32t (T11I)	c-41a	c32t (T11I)
Δ*mvaS*^ad^ 4	–	c40t (R14C)	c-41t	c40t (R14C)
Δ*mvaS*^ad^ 5	–	g41a (R14H)	g-52a	g41a (R14H)
Δ*mvaS*^ad^ 6	–	c40t (R14C)	c-41t	c40t (R14C)
Δ*mvaS*^ad^ 7	–	g41a (R14H)	g-52a	g41a (R14H)


*n.d., not determined.*

As shown in **Figure 1B** we also observed small colonies when the Δ*mvaS* mutants grew up after the long lag-phase. We wondered whether the small colonies were also mutated. Indeed, all the small colonies from five independent experiments carried a SNP in the *spx* gene but not in *drp35* (**Table 2**). The results prove that the adaptation of Δ*mvaS* to Δ*mvaS*^ad^ occurs stepwise via two successive mutations, the first occurring in *spx* and the second in the *drp35* gene.

### Phenotypic Characterization

For phenotypic characterizations and to elucidate the effect of the mutations on cell metabolism, SNPs were also cloned into the wild type, resulting in the strains HG001-*drp35^c-41t^*, HG001-*spx^T11I^* and HG001-*drp35^c-41t^/spx^T11I^.* In addition, a *spx* deletion mutant (Δ*spx*) was constructed.

### The Growth Rate Was Delayed in Δ*mvaS*^ad^

The doubling time of the single mutant, Δ*mvaS*-*spx^T11I^* was 29-times longer compared to the parent strain (**Table 3**). This shows that the Δ*mvaS*-*spx*^T11I^ mutant is able to grow but that the growth is severely impaired which is also indicated by the tiny colonies (**Figure 1B**). The adapted clone Δ*mvaS*^ad^ grew much better, due to the additional mutation in *drp35*. The doubling time was “only” 8-times higher compared to the parent strain and colonies on TSA were much larger (**Figures 1B,C**). The single mutant Δ*mvaS*-*drp35^c-41t^* was unable to grow in the absence of MVA, (**Figure 2B**). This result indicates that the mutation in *spx* occurs first during the adaptation and that a mutation occurring first in *drp35* cannot resuscitate growth.

**Table 3:** Growth rate of *S. aureus* strains.

Strain	Doubling time (h)	Factor of growth delay
**HG001**		
HG001	0.46 ± 0.03_a_	1
HG001-*drp35^c-41t^*	0.46 ± 0.01	1
HG001-*spx^T11I^*	0.50 ± 0.04	1.1
HG001-*drp35^c-41t^*/*spx^T11I^*	0.49 ± 0.03	1.1
Δ*spx*	0.82 ± 0.12	1.8
**Δ*mvaS***		
Δ*mvaS*^ad^	3.88 ± 0.39	8.4
Δ*mvaS*-*spx^T11I^*	13.4 ± 2.6	29.1
Δ*mvaS*-*drp35^c-41t^*/*spx^T11I^*	4.09 ± 0.72	8.9


*^*a*^Mean values ± SD of three independent growth curves.*

In HG001 the mutation *drp35^c-41t^* did not influence growth. However, *spx^T11I^* slightly increased the generation time 1.1-fold in both HG001-*spx^T11I^* and HG001-*drp35^c-41t^/spx^T11I^* (**Table 3**). However, complete deletion of *spx* reduced growth 1.8-fold. The strain Δ*mvaS*-*drp35^c-41t^/spx^T11I^* showed a similar doubling time than Δ*mvaS*^ad^. Further, both mutations had no influence on staphyloxanthin production. All knock-in strains in HG001 as well as Δ*spx* showed the typical orange color, whereas Δ*mvaS*^ad^ and Δ*mvaS*-*drp35^c-41t^/spx^T11I^* strains were white (Supplementary Figure S2), suggesting that the missing staphyloxanthin is due to the disrupted MVA pathway and not a result of the mutations. This makes sense as staphyloxanthin biosynthesis affords isoprenoid precursors (Pelz et al., 2005).

### Morphological Changes in Δ*mvaS*^ad^

**FIGURE 3:**
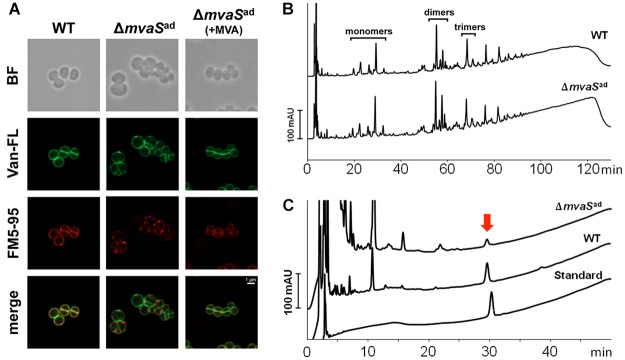
Comparison of the cell envelop of HG001 and Δ*mvaS*^ad^. **(A)** Fluorescence microscopy of HG001 and Δ*mvaS^ad^*. Cell membranes were stained with FM5-95 and PG was stained with Vancomycin, BODIPY^TM^ FL Conjugate (Van-FL). BF, bright field. **(B)** PG pattern of HG001 and Δ*mvaS^ad^* analyzed by RP-HPLC. v was isolated and digested with mutanolysin prior to analysis. **(C)** Amount of Undecaprenyl-phosphate (MW: 847.3) in HG001 and Δ*mvaS^ad^* cells determined by HPLC analysis. The amount was calculated to 287 ± 39 and 16.1 ± 1.7 nmol per gram of cell mass dry weight for HG001 and Δ*mvaS*^ad^, respectively. Each value is the mean ± SD of three individual experiments (*n* = 3). Undecaprenyl-monophosphate diammonium (MW: 881.4) was used as standard.

Already in bright field (BF) imaging it’s visible that the cells of the adapted Δ*mvaS*^ad^ clone were enlarged cocci and look swollen in contrast to the parent strain HG001 or Δ*mvaS*^ad^ grown in the presence of MVA (**Figure 3A**). Fluorescence staining with BODIPY^TM^ FL Vancomycin revealed that less vancomycin was bound in the cross wall of Δ*mvaS*^ad^ and spots with increased dye accumulation were visible. This suggests that less PG was present and that cross wall is largely deficient of uncross-linked PG. Membrane staining with FM5-95 revealed a weaker staining and dot-like structures (**Figure 3A**). In the merged image, membrane- and PG-stained areas do not really overlap. Overall, the microscopic studies suggest that Δ*mvaS*^ad^ has a severe deficiency in PG synthesis and that it is osmotic fragile as indicated by the blown-up cells.

The two mutations seem not to be the reason for the irregular morphology and cell wall staining. The strains HG001-*drp35^c-41t^*, HG001-*spx^T11I^* and HG001-*drp35^c-41t^/spx^T11I^* showed normal cell size and cell wall staining. Only the strains Δ*mvaS*^ad^ and Δ*mvaS*-*drp35^c-41t^/spx^T11I^* showed the abnormal morphology (Supplementary Figure S3).

### Peptidoglycan Structure Was Not Altered in Δ*mvaS*^ad^ but the Content of Bactoprenol Was Very Low

Because of the abnormal cell size and the irregular cell wall staining in Δ*mvaS*^ad^ we were interested whether the PG composition differed. PG was isolated from mid-exponential growth phase of Δ*mvaS*^ad^ and the parent strain HG001 and adjusted to the same OD_578_. The HPLC-profile of the mono-, di-, tri- and larger oligomers were not significantly altered in Δ*mvaS*^ad^ compared to the parent strain (**Figure 3B**).

As the primary structure of PG was apparently not altered we investigated whether undecaprenol, a follow product of MVA, could be formed by Δ*mvaS*^ad^. Undecaprenol is part of Lipid II and is needed to translocate PG precursors across the membrane. To answer this question, we extracted lipids from whole cells and the chloroform extract was analyzed for the presence of undecaprenol by RP-HPLC. We analyzed the phosphorylated undecaprenyl-phosphate (C_55_-P) and undecaprenyl-diphosphate (C_55_-PP). Chemical dephosphorylation with KOH, which converts C_55_-PP into C_55_-P, allowed the analysis and quantification of both forms in one peak. Commercial undecaprenyl-MPDA was used as a standard. The untreated standard showed one single and well-defined peak with a retention time of ∼ 30 min (**Figure 3C**). The chloroform extract of the parent strain showed that most of the substances eluted during the first 5 min and that C_55_-P could be detected very well. The small shift of the peak of the standard can be explained by the slightly higher molecular mass (881 g/mol compared to 847 g/mol). Interestingly, the same peak could also be found in Δ*mvaS*^ad^ cells, but the amount was 18-fold less compared to the parent strain (**Figure 3C**). The amount of phosphorylated undecaprenol in the parent strain was 282 nmol/g cell dry weight, while for Δ*mvaS*^ad^ it was only 16.1 nmol/g cell dry weight (**Table 4**). This result shows that Δ*mvaS*^ad^ can synthesize undecaprenol, whose synthesis normally needs IPP as precursor. Cloning of *drp35^c-41t^* or *spx^T11I^* into HG001 resulted in an increase in undecaprenol to 461 and 426 nmol/g cell dry weight, respectively. The strain Δ*mvaS*-*drp35^c-41t^/spx^T11I^* produced similar undecaprenol amounts as Δ*mvaS*^ad^.

**Table 4:** Amount of undecaprenol in different strains.

Strain	Amount of C_55_-P (P) [nmol/g cell dry weight]
**HG001**	
HG001	282 ± 33
HG001-*drp35^c-41t^*	461 ± 13
HG001-*spx^T11I^*	426 ± 113
HG001-*drp35^c-41t^*/*spx^T11I^*	372 ± 31
**Δ*mvaS***	
Δ*mvaS*^ad^	16.1 ± 1.7
Δ*mvaS*-*drp35^c-41t^*/*spx^T11I^*	20.8 ± 1.2


*Values are the mean ± SD of at least two independent Measurements.*

### Both Mutations in *spx* and *drp35* Enhance *drp35* Expression

**FIGURE 4:**
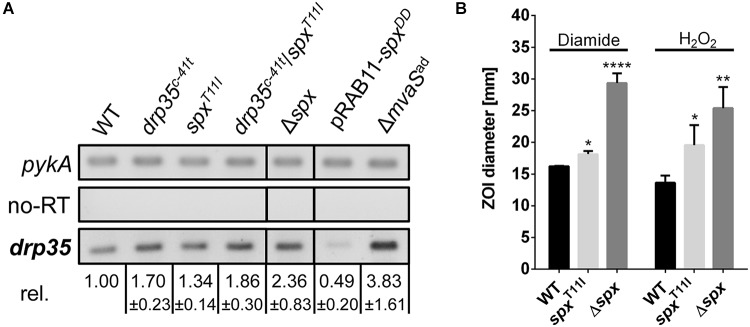
The point mutations *spx^T11I^* and *drp35^c-41t^* reduce the activity of Spx. **(A)** Expression level of *drp35* in HG001, its isogenic spx deletion mutant, its knock-in mutants *drp35^c-41t^, spx^T11I^ and drp35^c-41t^/spx^T11I^*, and during overexpression of a proteolysis resistant Spx *variant* (pRAB11*_spx^DD^*). Mean values ± SD are from two individual experiments. **(B)** Sensitivity of HG001, its isogenic spx mutant and HG001-*spx^T11I^* toward diamide and H_2_O_2_ determined by a filter disk diffusion assay. The ZOI values for diamide were 16.24 ± 0.05 mm (HG001), 18.16 ± 0.39 mm (HG001-*spx^T11I^*), and 29.36 ± 1.24 mm (HG001Δ*spx*). For H_2_O_2_ the values were 13.64 ± 0.92 (HG001), 19.58 ± 2.58 mm (HG001-*spx^T11I^*), and 25.40 ± 2.72 mm (Δ*spx*). Each value is the mean ± SD of three individual experiments (*n* = 3). ^∗^*p* < 0.05, ^∗∗^*p* < 0.01, and ^∗∗∗∗^*p* < 0.001 and ordinary one-way ANOVA for **(B)**.

The mutation in the promoter of *drp35* suggested an impact on gene expression. Also, we wondered whether the mutation in the transcriptional regulator gene *spx* influenced *drp35*-expression. In a semi-quantitative RT-PCR approach the expression of *drp35* in the exponential growth phase was determined in several strains. Relative to the parent strain, *drp35* was upregulated 1.7-fold in the knock-in mutant HG001-*drp35^c-41t^*, and 1.3-fold in HG001-*spx*^T11I^ (**Figure 4A**). The combination of both mutations (*drp35^c-41t^/spx*^T11I^) further increased *drp35* expression to 1.8-fold. The deletion of the *spx* gene resulted in a 2.4-fold higher *drp35* expression compared to the parent strain, whereas the over-expression of a proteolysis resistant Spx variant (pRAB11_*spx^DD^*, last two amino acids are aspartate) (Nakano et al., 2005; Wang et al., 2010) reduced the expression of *drp35* to 0.5-fold. In Δ*mvaS*^ad^ *drp35* was 3.8-fold higher expressed (**Figure 4A**).

### Comparative Transcriptome Analysis by Microarray Approach

By transcriptome analysis we tried to find out which genes in Δ*mvaS*^ad^ were differently expressed compared to the parent strain HG001. For that RNA was isolated from cells in the mid (*t*_1_) and late-exponential growth phase (*t*_2_). In total 181 genes were more than 2.0-fold differentially expressed at least at one of the time points. Fifty four genes were up-regulated in the adapted Δ*mvaS*^ad^, whereas 127 genes were down-regulated at *t*_1_ and/or *t*_2_ (Supplementary Tables S3, S4). Among the upregulated genes were the capsule biosynthesis genes and genes belonging to the cell wall stress stimulon (SAOUHSC_00560, *vraX*, SAOUHSC_00639, SAOUHSC_01173, SAOUHSC_02596, *cwrA*, and *drp35*) (Kuroda et al., 2003; Utaida et al., 2003). Among the down-regulated genes were those involved in nucleotide and amino acid metabolism, in regulation and particularly phage-related genes. It was remarkable that we didn’t find changes in the expression of the remaining genes of the MVAP or genes related to the synthesis of isoprenoids. The only exception was the upregulation of the glycosyl-4,4′-diaponeurosporenoate acyltransferase gene, which encodes the last enzyme in staphyloxanthin biosynthesis (Pelz et al., 2005). The lactonase-encoding *drp35* gene was upregulated 3.2- (*t*_1_) and 2.4-fold (*t*_2_), respectively.

### The Point Mutation in *spx* Decreased Spx Regulator Activity

Since Spx plays a key role during disulfide and oxidative stress (Nakano et al., 2003a), the sensitivity toward diamide and H_2_O_2_ was investigated. Filter disk diffusion assays with diamide and H_2_O_2_ were performed and the sensitivity was compared by measuring the diameter of the ZOI. Compared to the parent strain HG001, Δ*spx* was, as expected, much more sensitive toward both substances. Also HG001-*spx^T11I^* was significantly more sensitive, but not as sensitive as the deletion mutant (**Figure 4B**). These results, together with the results from the growth analysis and RT-PCR approach, show that the T11I-mutation in Spx reduces the activity of the regulator, but does not abolish it completely; the results also show that *spx* is an important regulator gene as its deletion has a severe impact on growth.

### Drp35 Is a Lactonase That Converts Mevalonolactone to Mevalonate

Drp35 is annotated as a lactonase and it has been shown that it can use the aromatic lactones dihydrocoumarine and 2-coumaranone as substrates (Morikawa et al., 2005). Using a colorimetric activity assay with purified strep-tagged Drp35 we found that MVL was rapidly hydrolyzed with a rate of 478 μmol/min/mg of protein. (**Table 5** and Supplementary Figure S4). The conversion rates of other tested lactones were at least 100-fold lower compared to MVL (**Table 5**). For some lactones we didn’t see any activity. This result suggests MVL as the physiological substrate of the lactonase Drp35.

**Table 5:** Conversion rate of different lactones.

Substrate	Conversion rate [μmol/min/mg of purified protein]
α-Angelica lactone	0.85 ± 0.27
α-Acetylbutyrolactone	3.6 ± 0.2
β-Butyrolactone	ND
L-Fucono-1,4-lactone	ND
Glucuronolactone	ND
δ-Hexalactone	2.5 ± 0.9
DL-Mevalonolactone	478 ± 68
γ-Octanoic lactone	ND
Pantolactone	0.002 ± 0.002
Undecanoic-δ-lactone	0.008 ± 0.006
γ-Valerolactone	0.016 ± 0.017
Whiskey lactone	0.026 ± 0.005
D-Xylono-1,4-lactone	0.018 ± 0.015


*Values are the mean ± SD of three independent measurements. ND, no activity detectable under these assay conditions.*

We also compared the mevalonolactonase activity in cell extracts of parent and mutant strains. Compared to the parent strain, HG001, the cell extract of strain HG001-*drp35^c-41t^* showed a 10-fold higher hydrolysis rate, and in the double mutant, HG001-*drp35^c-41t^/spx^T11I^*, the activity was even 14-fold higher compared to the parent strain (**Table 6**). The mevalonolactonase activity of cell extracts of strain HG001-*spx^T11I^* was slightly increased (1.6-fold) compared to HG001. Cell extracts of the adapted mutant, Δ*mvaS*^ad^, and the complemented non-adapted mutant, Δ*mvaS*-*drp35^c-41t^/spx^T11I^*, had a 18- and 10-fold, respectively, higher activity than the parent strain HG001; indicating that the mutation in *drp35^c-41t^* contributes more to lactonase activity than the mutation in *spx^T11I^*.

**Table 6:** Hydrolysis rate of MVL by cell extracts.

Cell extract	conversion rate [μmol MVL/min/mg of total protein]
**HG001**	
HG001	0.022 ± 0.009
HG001-*drp35^c-41t^*	0.234 ± 0.104
HG001-*spx^T11I^*	0.035 ± 0.012
HG001-*drp35^c-41t^* /*spx^T11I^*	0.310 ± 0.041
Δ*spx*	0.105 ± 0.081
**Δ*mvaS***	
Δ*mvaS*^ad^	0.419 ± 0.201
Δ*mvaS*- *drp35^c-41t^*/*spx^T11I^*	0.233 ± 0.043


*Values are the mean ± SD of three independent measurements.*

### Growth of Δ*mvaS*^ad^ Is Inhibited by 6-Fluoromevalonate

**FIGURE 5:**
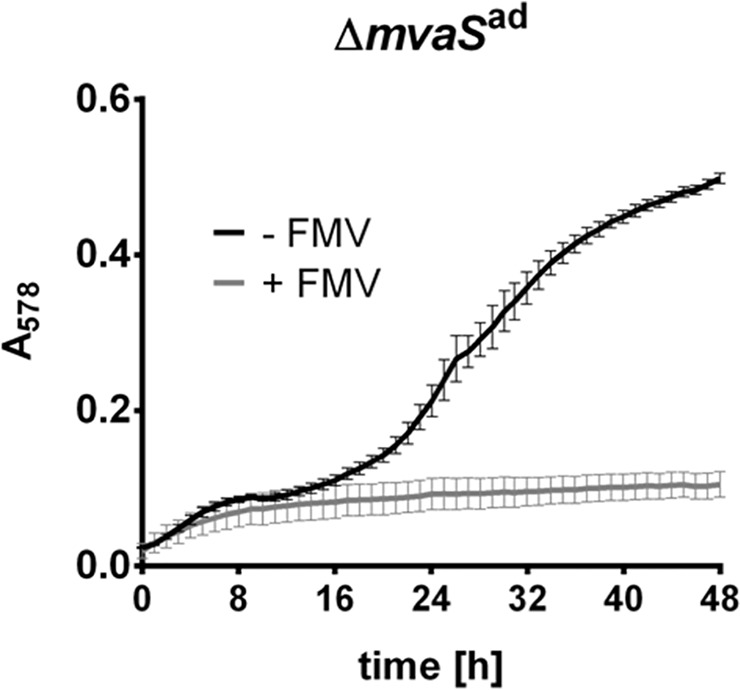
Growth of Δ*mvaS*^ad^ is inhibited by 6-Fluoromevalonate. Growth of Δ*mvaS*^ad^ in the presence or absence of 6-Fluoromevalonate (FMV, inhibitor of diphosphomevalonate decarboxylase MvaD). Cells were grown in the presence of 5% DMSO and 150 μg/ml FMV or only 5% DMSO (control). Growth was followed in a Tecan Infinite M200 microplate reader at 37°C with shaking intervals for 48 h. Absorbance at 578 nm was measured every hour. Each data point represents the mean value ± SD of three independent experiments (*n* = 3).

To investigate whether the MVAP, downstream of MvaS, is still functional, we grew the Δ*mvaS*^ad^ in the presence of FMV. FMV is an inhibitor of the diphosphomevalonate decarboxylase MvaD, which is the last enzyme in the MVAP. In the presence of FMV Δ*mvaS*^ad^ was not able to grow, indicating that the lower MVAP is still needed in the mutant (**Figure 5**).

### Virulence of Δ*mvaS*^ad^ Is Decreased

**FIGURE 6:**
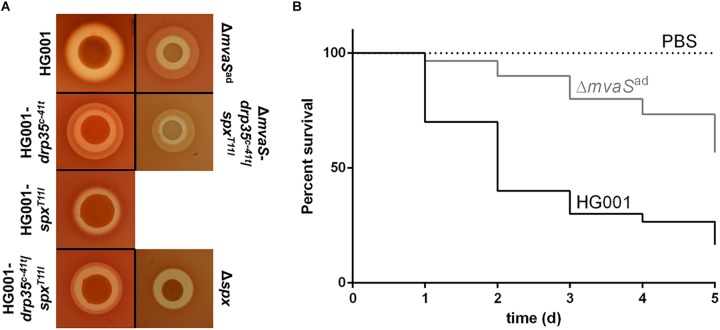
The adapted mutant shows differences in the hemolytic activity and reduced virulence. **(A)** Overnight cultures of HG001, HG001-*drp35^c-41t^*, HG001-*spx^T11I^*, HG001-*drp35^c-41t^/spx^T11I^*, Δ*spx*, Δ*mvaS*^ad^, and Δ*mvaS-*-*drp35^c-41t^/spx^T11I^*, were diluted to OD_578_ 1 and dropped on sheep blood agar plates. Plates were incubated at 37°C for 72 h. **(B)** Larvae of *Galleria mellonella* were infected with 5 × 10^6^ cells of HG001 and Δ*mvaS*^ad^. For each strain 30 larvae separated into three individual experiments were used. Larvae were incubated at 37°C for 5 days and survival was checked every 24 h. The virulence of Δ*mvaS*^ad^ is significantly reduced with *p* < 0.0001, by log-rank (Mantel-Cox) test.

To test whether the adapted mutant is pathogenic the hemolytic activity as well as the ability to cause infections was tested. A drop test on sheep agar plates showed that Δ*mvaS*^ad^ still lysed erythrocytes (**Figure 6A**). However the appearance of the hemolytic zone was different. The parent strain HG001 showed only a clear halo, whereas Δ*mvaS*^ad^ showed in addition a turbid halo surrounding the clear halo. Wide clear zones are indicative for α-hemolysin and turbid halos for β-hemolysin activity (Adhikari and Novick, 2008). The strains HG001-*drp35^c-41t^*, HG001-*drp35^c-41t^*/*spx^T11I^* and Δ*mvaS*-*drp35^c-41t^/spx^T11I^* also produced the outer turbid zone, whereas HG001-*spx^T11I^* and Δ*spx* did not. Unexpectedly, this result suggests that the expression of ß-hemolysin is somehow affected by the overexpression of *drp35* and not by the reduced activity of Spx^T11I^.

The *Galleria mellonella* infection model showed that the virulence of Δ*mvaS*^ad^ is significantly reduced compared to the parent strain (**Figure 6B**). Larvae infected with the parent strain showed a survival rate of only 17% (5 out of 30 larvae), with more than 50% dead larvae within the first 2 days. The survival rate of larvae infected with the adapted mutant amounted to 57% (17 out of 30). In the first 2 days only 10% percent of the larvae died.

## Discussion

The synthesis of isoprenoids is essential in all living organisms. In *S. aureus* the MVAP is the only route to synthesize the universal isoprenoid-precursor isopentenyl-pyrophosphate, which makes the MVA pathway a potential target for antimicrobial compounds. Recently, we created a Δ*mvaS* deletion mutant in *S. aureus* and, as expected, the mutant was unable to grow in medium lacking MVA (Yu et al., 2013). However, to our surprise, after prolonged cultivation for 4–6 days the mutant started to grow.

Here, we addressed the question what adaptation processes took place in the prolonged lag-phase that allowed the Δ*mvaS* mutant to grow. As the Δ*mvaS* mutant has adapted from auxotrophic to prototrophic phenotype we referred the adapted mutant as Δ*mvaS*^ad^. Comparative genome sequencing of the parent strain HG001 and the adapted mutant Δ*mvaS*^ad^ revealed, that the mutant aquired two point mutations in different genes, namely *spx* and *drp35*.

### Only Specific Mutations Lead to Mevalonate Prototrophy in Δ*mvaS*^ad^

We wondered whether the mutational adaptation of Δ*mvaS* to MVA-prototrophy in Δ*mvaS*^ad^ could be repeated. Therefore, we carried out the adaptation procedure in total seven times. In all cases we found the mutations in a close range of *spx* and *drp35* (**Table 2**). This indicates that only a few specific mutations lead to the adaptation of the mutant.

This resembles on the selection of antibiotic resistant mutants such as ciprofloxacin or rifampicin (RIF). Ciprofloxacin and other quinolones target the A subunit of DNA gyrase and selection of spontaneous single-step resistance mutants of *E. coli* and other bacteria were mostly *gyrA* mutations (Hooper et al., 1987). Resistance to RIF is nearly always due to a mutation in the β-subunit of bacterial RNA polymerase (RNAP) (Goldstein, 2014). The difference between these single-step resistance mutants involved in antibiotic resistance to the adaptation of Δ*mvaS*^ad^ to MVA-prototrophy is that in our case two mutations, instead of only one mutation, are necessary.

### The Adaptation of Δ*mvaS* to Mevalonate Prototrophy Is Based on Two Sequential Mutations in Different Genes

The question was whether the mutations in *spx* and *drp35* occurred in a special order or simultaneously. A simultaneous mutation is completely unlikely as the frequency would be extremely low (multiplication of the frequency of each single mutation). So the question remained which mutation appears first or if it is a random event. When Δ*mvaS* started to grow after the lag-phase of 4–6 days we observed always a mixture of very small and larger colonies on agar plates (**Figure 1B**). All the small colonies we investigated carried only one mutation, namely in *spx*. All the large colonies carried the double mutations in both *spx* and *drp35* (**Figures 1B,C** and **Table 2**). We never found colonies with a mutation in *drp35* only. This result indicates that the first mutation is in the regulator gene *spx* following the second mutation in *drp35*. The mutations in this order make sense, because, the *spx^T11I^* mutation allows already slight growth, which facilitates the development of the second mutation. Growth is necessary for the second mutation, as mutations occur mainly during DNA replication (Luria and Delbruck, 1943; Foster, 2004).

### The Mutation *spx^T11I^* Reduced the Regulator Activity of Spx

Spx is described as a global transcriptional regulator in *S. aureus*, which is mainly active under stress conditions, but also contributes to cell fitness under non-stress conditions (Pamp et al., 2006). The *Bacillus subtilis* specific Spx does not bind directly to DNA, but influences gene transcription by interacting with the C-terminal domain of the α-subunit (αCTD) of the RNA Polymerase, a common target for transcriptional activators (Nakano et al., 2003b). The point mutation in *spx* is located in the coding region and caused an amino acid exchange from threonine to isoleucine at position 11 (T11I). The amino acid substitution is located in the so-called C-X-X-C motif, which mediates enzyme activity by the reduction of the cysteine residues. We assume that the change in this motif affects the general activity of Spx, in both positive and negative way. To see whether oxidative stress tolerance is affected, we introduced the mutation in the parent strain, HG001-*spx^T11I^*. Indeed, this mutation made HG001-*spx^T11I^* more sensitive against oxidative stress triggered by the thiol-reactive chemical diamide and by H_2_O_2_. Deletion of *spx* further increased the sensitivity, indicating, that the activity of Spx^T11I^ is reduced but not completely abolished.

Spx is a negative regulator of *drp35* expression, as verified by several observations: In HG001-*spx^T11I^ drp35* was 1.7-fold up-regulated and 2.4-fold in Δ*spx*. When *spx* was over-expressed in HG001 (pRAB11*spx^DD^*) *drp35* expression was repressed. Furthermore, the value for mevalonolactonase activity of strain HG001-*spx^T11I^* was in between the values of Δ*spx* and HG001. All our results suggest that the activity of Spx^T11I^ is not abolished, but severely alters the regulator function. But why this reduced activity is beneficial for the mutant is still not completely clear.

### Drp35 Is Overexpressed and Shows Mevalonolactone Lactonase Activity

The point mutation of *drp35^c-41t^* in Δ*mvaS*^ad^ was located at position -41, which is in the so-called UP (upstream)-element of the -35-region of the core-promoter. Comparative transcriptome analysis showed that *drp35* is up-regulated two to three times in Δ*mvaS*^ad^, indicating that this mutation increased *drp35* expression, which could be confirmed by RT-PCR. UP-elements are in general AT-rich and stimulate gene transcription (Petho et al., 1986; Estrem et al., 1998). In *drp35^c-41t^* and the other adapted mutants, the AT-content of the UP-element is further increased and we assume that this is the reason of the transcriptional up-regulation of *drp35^c-41t^*. Activity tests showed that the hydrolysis of MVL to MVA can be carried out by Drp35 much more efficient than the hydrolysis of other substrates. This indicates that MVL might be the physiological substrate of the lactonase Drp35. The higher MVL hydrolyzing activity of cell extracts from the adapted mutant and strains harboring the mutations showed that the up-regulation of *drp35* results in a higher protein amount in the cells.

Also, we showed that the adapted mutant Δ*mvaS*^ad^ can produce a small amount of undecaprenol, which is essential for cell wall biosynthesis. Both mutations seem to influence undecaprenol biosynthesis, as cloning of *drp35^c-41t^* or *spx^T11I^* into HG001 increased the undecaprenol level.

### Only the Upper Part of the MVA Pathway Is Bypassed

In general, a decreased activity of the transcriptional regulator Spx and the overproduction of the lactonase Drp35 is needed to regain cell proliferation of Δ*mvaS*^ad^. However, the growth of Δ*mvaS*^ad^ is slow and does not reach the level of growth when the medium contains MVA. The cell wall appears to be fragile and disordered and the blown-up cells indicate that the cells cannot withstand osmotic stress. Nevertheless, the mutations in *drp35^c-41t^* and *spx^T11I^* seem to allow a limited bypass of the upper MVA pathway to MVA. Bypassing essential metabolic pathways is not unusual in bacteria. For example, glutamate and proline auxotrophic mutants of *B. subtilis* can revert to prototrophy by suppressor mutations or amplification of specific genomic loci (Zaprasis et al., 2014; Dormeyer et al., 2017).

There are several reasons why we think that MVA is the bypass product. First, we investigated whether genes downstream of *mvaS* could be deleted in Δ*mvaS*^ad^. We were able to delete *mvaA* without affecting the growth (Supplementary Figure S5) but not the *mvaK1* gene, suggesting that enzymes downstream of MVA are essential and that there is not a completely different pathway, which was activated (see Supplementary Figure S1A). Second, Δ*mvaS*^ad^ is not able to grow in the presence of FMV (inhibitor of MvaD). Furthermore, when examining the location of *drp35* in various staphylococcal genomes we found that in some species, like *S. xylosus, drp35* is positioned directly next to *mvaS* and *mvaA.* In *S. sciuri* strain FDAARGOS_285 it is located directly upstream and in the same orientation of the mvaK1/D/K2 operon indicating an involvement of Drp35 in the MVA pathway (Supplementary Figure S1B and Supplementary Table S5). Only in *S. aureus* and some other species it is dislocated. Finally, it is remarkable that Drp35 shows the highest lactonase activity with MVL.

The upper MVA pathway, in particular MvaA, is considered as a potential target for the development of new antibiotics (Wilding et al., 2000). Therefore, we were interested if the adapted mutant, who seems to have a bypass for the upper MVA pathway, is still pathogenic. If so, the theory of targeting MvaA would be questionable, because challenging *S. aureus* with such an antibiotic would probably lead to the emergence of resistant and virulent mutants. However, in a *Galleria mellonella* infection model it turned out that the virulence of the adapted mutant is greatly reduced, which might be due to the missing staphyloxanthin and the more fragile cell wall. Additionally, the growth on sheep blood agar showed that *drp35^c-41t^* influenced hemolysin production.

Altogether, we showed here that a MVA auxotrophic *mvaS* mutant can revert to prototrophy by acquiring two point mutations in the genes *spx* and *drp35*. The mutations led to a decreased regulator activity of Spx and to an increased expression of the lactonase Drp35. Based on these mutations the adapted mutant, Δ*mvaS*^ad^, seems to be able to synthesize sufficient MVA to produce small amounts of the lipid carrier undecaprenol. However, the amount of produced MVA is not high enough to allow a fast and proper cell wall synthesis or to synthesize staphyloxanthin. The adapted Δ*mvaS*^ad^ mutant appears to have a rescue pathway that allows cell proliferation in the absence of the upper MVA pathway. We could not uncover this bypass completely. Nevertheless this study illustrates once again how adaptive the bacterial genome is. Like a lifeless creature a subpopulation is able to rise again by genetic adaptation. Interestingly, the adaptation includes always the two point mutations in *spx* and *drp35* and always in the order *spx* first and *drp35* second, a classical case of adaptive mutation.

## Author Contributions

FG and SR designed the study. SR, PE, AL, MN, JS, JO, PS, PF, and FG designed the experiments. SR performed most of the experiments. E-JB and PF performed transcriptome analysis. MN performed PG analysis. BB and CS performed whole genome sequencing and analysis and PS performed fluorescence microscopy. PE contributed to proofreading. FG and SR wrote the manuscript.

## Conflict of Interest Statement

The authors declare that the research was conducted in the absence of any commercial or financial relationships that could be construed as a potential conflict of interest. The reviewer FC and handling Editor declared their shared affiliation.

## Footnotes

1 http://www.ncbi.nlm.nih.gov/geo/

## Abbreviations

DMAPP: dimethylallyl pyrophosphate
FMV: 6-Fluoromevalonate
IPP: isopentenyl pyrophosphate
MEP: 2C-methyl-D-erythritol 4-phosphate pathway
MVA: mevalonate
MVL: mevalonolactone
MVAP: mevalonate pathway
PG: peptidoglycan
