# Supplementary material for: Genetic Adaptation of a Mevalonate Pathway Deficient Mutant in Staphylococcus aureus
Source: Front Microbiol. 2018 Jul 12;9:1539. doi: 10.3389/fmicb.2018.01539 (PMC6052127; doi:10.3389/fmicb.2018.01539)
Supplement: Supplementary file 1 [file Image_1.PDF]

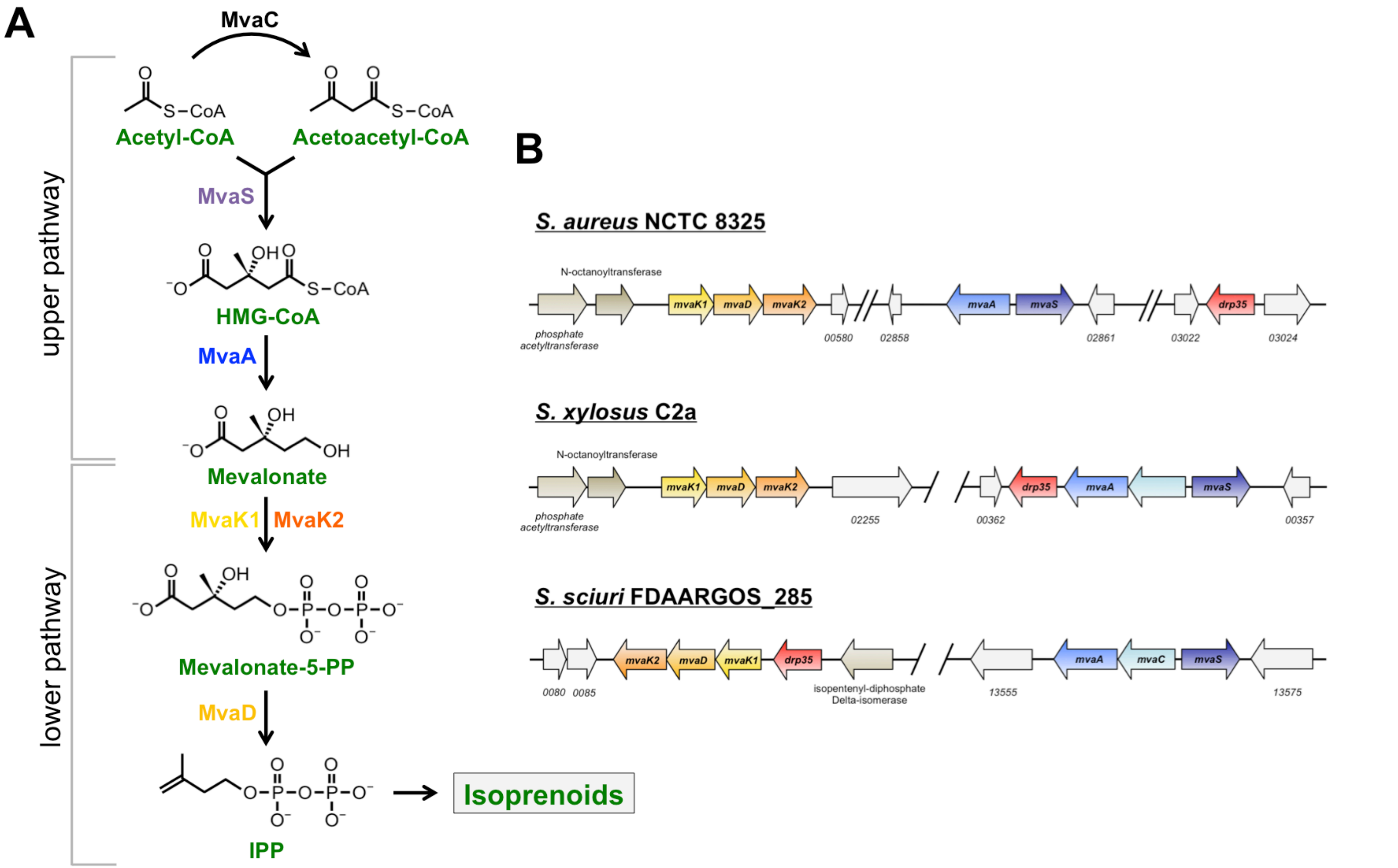
A
MvaC
Acetyl-CoA
Acetoacetyl-CoA
MvaS
HMG-CoA
MvaA
Mevalonate
MvaK1
MvaK2
Mevalonate-5-PP
MvaD
IPP
Isoprenoids
upper pathway
lower pathway
B
S. aureus NCTC 8325
N-octanoyltransferase
phosphate acetyltransferase
mvaK1
mvaD
mvaK2
00580
02858
mvaA
mvaS
02861
03022
drp35
03024
S. xylosus C2a
N-octanoyltransferase
phosphate acetyltransferase
mvaK1
mvaD
mvaK2
02255
00362
drp35
mvaA
mvaS
00357
S. sciuri FDAARGOS_285
0080
0085
mvaK2
mvaD
mvaK1
drp35
isopentenyl-diphosphate Delta-isomerase
13555
mvaA
mvaC
mvaS
13575

Fig S1

Figure S1: Schematic representation of the mevalonate pathway and the organization of the corresponding genes. (A) Schematic representation of the mevalonate pathway in *S. aureus.* Two molecules Acetyl-CoA are condensed to Acetoacetyl-CoA by the Acetyl-CoA acetyltransferase MvaC. Hydroxymethoylglutaryl-CoA (HMG-CoA) is build by the condensation of Acetoacetyl-CoA and another molecule of Acetyl-CoA by the Hydroxymethoylglutaryl-CoA synthase MvaS. HMG-CoA is then reduced to mevalonate by the Hydroxymethoylglutaryl-CoA reductase MvaA. Mevalonate gets doubly phosphorylated by the two kinases MvaK1 and MvaK2 and decarboxylazed to isopentenyl diphosphate (IPP) by the mevalonate diphosphate decarboxylase MvaD. IPP is used as the common precursor for the synthesis of all isoprenoids. **(B)** Chromosomal location of the lactonase gene *drp35* in *S. aureus* NCTC 8325, *S. xylosus* C2a and *S. sciuri* FDAARGOS_285. The genes of the upper MV pathway *mvaS* and *mvaA* are located next to each other in opposite orientation. In *S. xylosus* C2a *drp35* is located next to these genes. In *S. sciuri* FDAARGOS_285 *drp35* is located upstream of *mvaK1/D/K2. Drp35* of *S. aureus* is dislocated from the MVA pathway genes.
