# Supplementary material for: Genetic Adaptation of a Mevalonate Pathway Deficient Mutant in Staphylococcus aureus
Source: Front Microbiol. 2018 Jul 12;9:1539. doi: 10.3389/fmicb.2018.01539 (PMC6052127; doi:10.3389/fmicb.2018.01539)
Supplement: Supplementary file 2 [file Image_2.PDF]

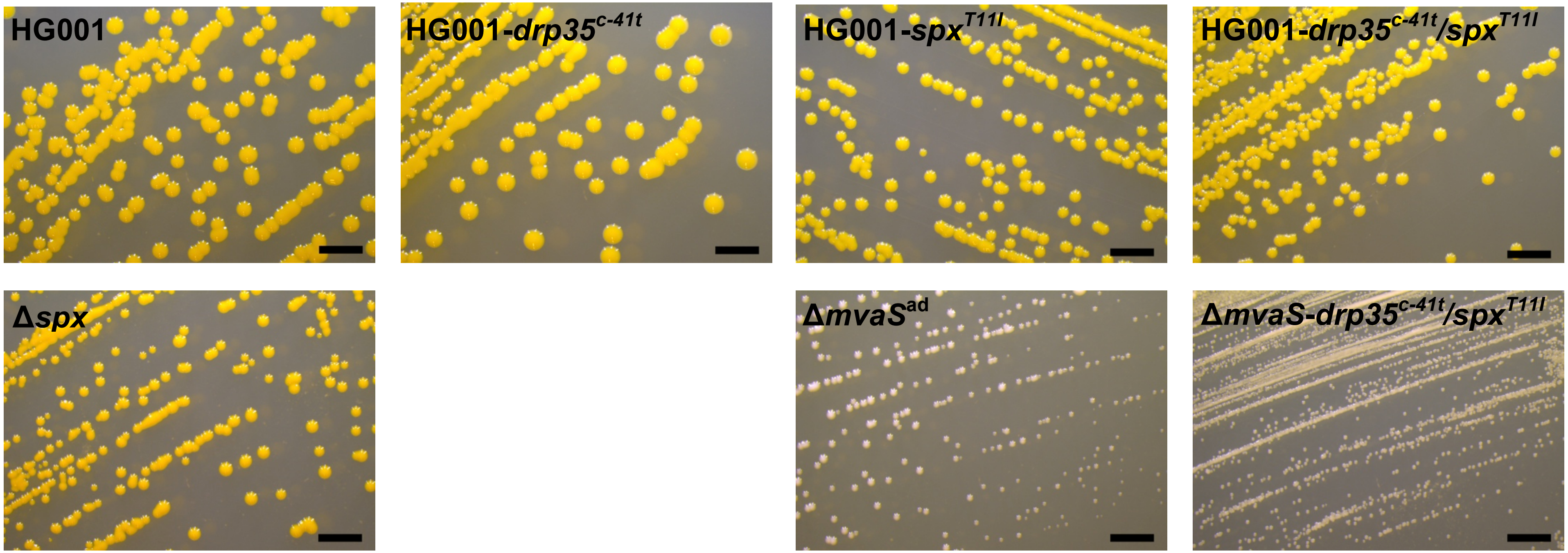

Figure S2: The two SNPs does not influence staphyloxanthin production . The strains HG001, HG001-*drp35*$^{c\text{-}41t}$, HG001-*spx*^{T11I}, HG001-*drp35*$^{c\text{-}41t}$/*spx*^{T11I}, Δ*spx*, Δ*mvaS*^{ad}, Δ*mvaS-spx*^{T11I}, and Δ*mvaS-drp35*$^{c\text{-}41t}$/*spx*^{T11I} were grown on TSA.
