# Supplementary material for: Genetic Adaptation of a Mevalonate Pathway Deficient Mutant in Staphylococcus aureus
Source: Front Microbiol. 2018 Jul 12;9:1539. doi: 10.3389/fmicb.2018.01539 (PMC6052127; doi:10.3389/fmicb.2018.01539)
Supplement: Supplementary file 3 [file Image_3.PDF]

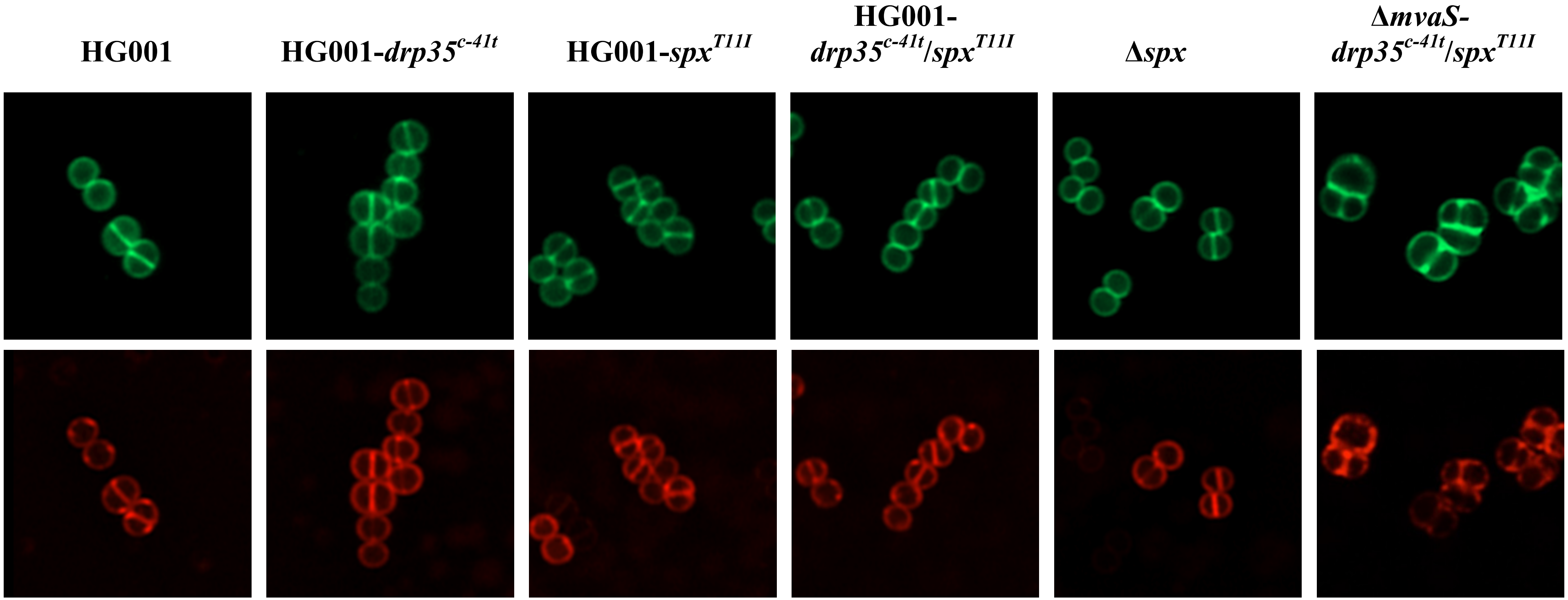

Figure S3: The two SNPs does not influence cell size, cell wall and membrane staining. The strains HG001, HG001-$drp35^{c\text{-}41t}$, HG001-spx^{T11I}, HG001-$drp35^{c\text{-}41t}/spx^{T11I}$, Δ*spx*, and Δ*mvaS*-$drp35^{c\text{-}41t}/spx^{T11I}$ were grown to mid-log phase and labeled with BODIPY™ FL Conjugate and FM5-95.
