# Supplementary material for: Genetic Adaptation of a Mevalonate Pathway Deficient Mutant in Staphylococcus aureus
Source: Front Microbiol. 2018 Jul 12;9:1539. doi: 10.3389/fmicb.2018.01539 (PMC6052127; doi:10.3389/fmicb.2018.01539)
Supplement: Supplementary file 4 [file Image_4.PDF]

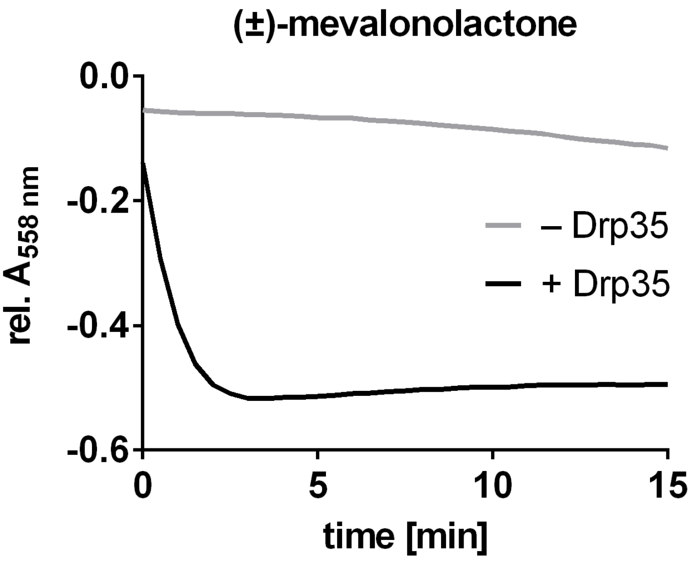

Figure S4: Drp35 converts mevalonolactone to mevalonate. Hydrolysis of mevalonolactone to mevalonate in the presence and absence of Drp35 monitored by the reduction of the Absorbance at 558 nm. Each data point is the mean value ± SD of three independent experiments. For aesthetic reasons error bars were excluded from the graph.
