# Supplementary material for: Genetic Adaptation of a Mevalonate Pathway Deficient Mutant in Staphylococcus aureus
Source: Front Microbiol. 2018 Jul 12;9:1539. doi: 10.3389/fmicb.2018.01539 (PMC6052127; doi:10.3389/fmicb.2018.01539)
Supplement: Supplementary file 5 [file Image_5.PDF]

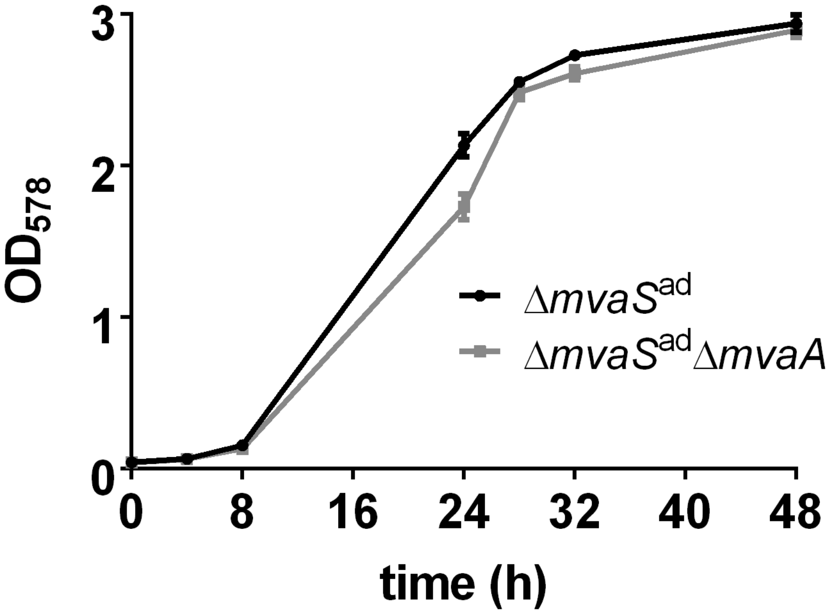

Figure S5: Deletion of *mvaA* does not influence growth of Δ*mvaS*^{ad} . Growth of Δ*mvaS*^{ad}Δ*mvaA* and Δ*mvaS*^{ad} was followed for 48 h. Each data point is the mean value ± SD of three independent experiments.
