# Supplementary material for: Genetic Adaptation of a Mevalonate Pathway Deficient Mutant in Staphylococcus aureus
Source: Front Microbiol. 2018 Jul 12;9:1539. doi: 10.3389/fmicb.2018.01539 (PMC6052127; doi:10.3389/fmicb.2018.01539)
Supplement: Supplementary file 6 [file Table_1.DOCX]

**Table S1:** strains and plasmids used in this study

| **Strain or plasmid** | **Description** | **Source of Reference** |
| --- | --- | --- |
| **strain** |  |  |
| *Escherichia coli* DC10B | *E. coli* K12, Δdcm mutant | (Monk et al., 2012) |
| *Staphylococcus aureus* HG001 | *S. aureus* NCTC 8325 derivative, *rsbU* repaired | (Herbert et al., 2010) |
| HG001Δ*mvaS* | *mvaS* deletion mutant, grows only in the presence of mevalonate | (Yu et al., 2013) |
| HG001Δ*mvaS*^ad^ | *mvaS* suppressor mutant | this study |
| HG001Δ*mvaS*^ad^Δ*mvaA* | deletion mutant of *mvaA* in Δ*mvaS*^ad^ | this study |
| HG001*spx*^T11I^ | harbors SNP in *spx* | this study |
| HG001*drp35^c-41t^*/*spx*^T11I^ | harbors SNP in *spx* and in promoter of *drp35* | this study |
| HG001Δ*spx* | *spx* deletion mutant | this study |
| HG001pRAB11-*spx*^DD^ | Harbours a plasmid for over-expression of a proteolysis-resistant Spx variant | this study |
| Δ*mvaS-drp35^c-41t^* | harbors SNP in promoter of *drp35* | this study |
| Δ*mvaS-spx*^T11I^ | harbors SNP in *spx* | this study |
| Δ*mvaS-drp35^c-41t^*/*spx*^T11I^ | harbors SNP in *spx* and in promoter of *drp35* | this study |
| **plasmid** |  |  |
| pBASE6 | Plasmid for construction of deletion and knock-in mutants | (Bae and Schneewind, 2006) |
| pBASE_*drp35^c-41t^*-KI | knock-in plasmid for SNP *drp35^c-41t^* | this study |
| pBASE_*spx*^T11I^-KI | knock-in plasmid for SNP *spx^T11I^* | this study |
| pBASE_*drp35*-KO | plasmid for deletion of *drp35* | this study |
| pBASE_*spx*-KO | plasmid for deletion of *spx* | this study |
| pBASE_*mvaA*-KO | plasmid for deletion of *mvaA in* Δ*mvaS* | this study |
| pBASE_*mvaK1*-KO | plasmid for deletion of *mvaK1* | this study |
| pRAB11 | Over-expression plasmid, ATc-inducible promoter | (Helle et al., 2011) |
| pRAB11-*spx^DD^* | Over-expression of a proteolysis-resistant Spx variant | this study |
| pPTtuf | Over-expression plasmid, tuf-promoter (constitutively) | (Popella et al., 2016) |
| pPT-tuf_*drp35*-strep | Over-expression of a strep-tagged Drp35 variant | this study |

**References**

Bae, T., and Schneewind, O. (2006). Allelic replacement in *Staphylococcus aureus* with inducible counter-selection. *Plasmid* 55(1)**,** 58-63. doi: 10.1016/j.plasmid.2005.05.005.

Helle, L., Kull, M., Mayer, S., Marincola, G., Zelder, M.E., Goerke, C., et al. (2011). Vectors for improved Tet repressor-dependent gradual gene induction or silencing in *Staphylococcus aureus*. *Microbiology* 157(Pt 12)**,** 3314-3323. doi: 10.1099/mic.0.052548-0.

Herbert, S., Ziebandt, A.K., Ohlsen, K., Schafer, T., Hecker, M., Albrecht, D., et al. (2010). Repair of global regulators in *Staphylococcus aureus* 8325 and comparative analysis with other clinical isolates. *Infect Immun* 78(6)**,** 2877-2889. doi: 10.1128/IAI.00088-10.

Monk, I.R., Shah, I.M., Xu, M., Tan, M.W., and Foster, T.J. (2012). Transforming the untransformable: application of direct transformation to manipulate genetically *Staphylococcus aureus* and *Staphylococcus epidermidis*. *MBio* 3(2). doi: 10.1128/mBio.00277-11.

Popella, P., Krauss, S., Ebner, P., Nega, M., Deibert, J., and Götz, F. (2016). VraH Is the Third Component of the *Staphylococcus aureus* VraDEH System Involved in Gallidermin and Daptomycin Resistance and Pathogenicity. *Antimicrob Agents Chemother* 60(4)**,** 2391-2401. doi: 10.1128/AAC.02865-15.

Yu, W., Leibig, M., Schafer, T., Bertram, R., Ohlsen, K., and Götz, F. (2013). The mevalonate auxotrophic mutant of *Staphylococcus aureus* can adapt to mevalonate depletion. *Antimicrob Agents Chemother* 57(11)**,** 5710-5713. doi: 10.1128/AAC.00726-13.
