# Supplementary material for: Genetic Adaptation of a Mevalonate Pathway Deficient Mutant in Staphylococcus aureus
Source: Front Microbiol. 2018 Jul 12;9:1539. doi: 10.3389/fmicb.2018.01539 (PMC6052127; doi:10.3389/fmicb.2018.01539)
Supplement: Supplementary file 7 [file Table_2.DOCX]

**Table S2:** Oligonucleotides used in this study

| **Primer name** | **Sequence** | **Purpose** |
| --- | --- | --- |
| **cloning** |  |  |
| *drp35*-KI_fwd | ccgggctagcgcgcagatctCACATGGTTTGCCATCAAAC | Cloning of  pBASE_*drp35^c-41t^*-KI |
| *drp35*-KI_rev | gcttgatatcgtcgacagatctCAAAGATGTTAAATTCGAAGCATC |  |
| up-*drp35*_fwd | ccgggctagcgcgcagatctCACATGGTTTGCCATCAAAC | Cloning of pBASE_*drp35*-KO |
| up-*drp35*_rev | ggtaggtcATCTATCTATTTTATCGGTCTAGTG |  |
| down-*drp35*_fwd | atagatagatGACCTACCTGAATGAAAGG |  |
| down-*drp35*_rev | gcttgatatcgtcgacagatCTATAAAAATGATAATAATAGTCAGATTTATCTC |  |
| *drp35*-OE_fwd | gggtaccgaggctcgaattcaattaggaggtattaattATGATGTCACAACAAGATTTAC | Cloning of  pPT-tuf_*drp35*-strep |
| *drp35*-strep_rev | agtctattattaagtactgaattcTTATTTTTCAAATTGTGGATGTGACCATTGAAACTGAAAACTTTGATGAC |  |
| *drp35*-seq_fwd | AGCGTCAATACGCATATTTGTC | Sequencing of *drp35* |
| *drp35*-seq_rev | AAACTGGTGGCGTAATGCTAGG |  |
| *spx*-KI_fwd | ccgggctagcgcgcagatCTAGTACCTCTTTATTTTATCTCTTAC | Cloning of pBASE_*spx*^T11I^-KI |
| *spx*-KI_rev | gcttgatatcgtcgacagatcTAGTGCGTTGCTTAATTAATATC |  |
| up-*spx*_fwd | ccgggctagcgcgcagatCTAGTACCTCTTTATTTTATCTCTTAC | Cloning of pBASE_*spx*-KO |
| up-*spx*_rev | gtaaagtatgCTTAAAATTTAGTTATAGATCAAGAAAAAAC |  |
| down-*spx*_fwd | taaattttaagCATACTTTACTAAAAGATATGTCAAGC |  |
| down-*spx*_rev | gcttgatatcgtcgacagatctCATAGTGCGTTGCTTAATTAATATC |  |
| *spx*-OE_fwd | gtatgatggtaccgttaacagatctaggaggtattaattATGGTAACATTATTTACTTCACC | Cloning of pRAB11_*spx^DD^* |
| *spx^DD^*-OE_rev | ggccagtgaattcgagctcagatcTTAGTCATCCATACGTTGTG |  |
| *spx-seq_fwd* | ATGTAAGATGTGTTAGAATATACG | Sequencing of *spx* |
| *spx-seq_rev* | GTTAAAGCATTAAAGCACTTTC |  |
| up-*mvaA*_fwd | ggtacccgggctagcgcgcagatctGTACTTTTCCACTAATAGTAGGTG | Cloning of pBASE_mvaA-KO |
| up-*mvaA*_rev | gttgtctaatTACTATTTTTACCACAAAATAACGTC |  |
| down-*mvaA*_fwd | taaaaatagtaATTAGACAACAATAGTAAATGGC |  |
| down-*mvaA*_rev | caagcttgatatcgtcgacagatctGAGCATTTATAACAAAAAGTATAATCC |  |
| up-*mvaK1*_fwd | ccgggctagcgcgcagatCTGGATTAGAACCTATGCAATC | Cloning of pBASE_mvaK1-KO |
| up- *mvaK1*_rev | taacctcctaaTCTTGTCATGTCAATATCACC |  |
| down- *mvaK1*_fwd | atgacaagaTTAGGAGGTTAATGCGTTG |  |
| down- *mvaK1*_rev | gcttgatatcgtcgacagatCTGAATCATTTTATCTCTTCCTTAC |  |
| **RT-PCR** |  |  |
| pykA | AGCTGAAGCAGCCCAAGATT |  |
| pykA | TACGTGCCGTTGAACCACTT |  |
| *drp35*-RT1 | ACTAGGAGGCGTTTATTATG |  |
| *drp35*-RT2 | TAGCTCCAAATGGTTGTATC |  |
