# Supplementary material for: Genetic Adaptation of a Mevalonate Pathway Deficient Mutant in Staphylococcus aureus
Source: Front Microbiol. 2018 Jul 12;9:1539. doi: 10.3389/fmicb.2018.01539 (PMC6052127; doi:10.3389/fmicb.2018.01539)
Supplement: Supplementary file 10 [file Table_5.DOCX]

**Table S5:** Adjacent genes of *drp35*-orthologues in different staphylococcal species.

| **Species and strain** | **Gene(s) upstream of**  ***drp35*-homologue** | **Gene(s) downstream of**  ***drp35*-homologue** |
| --- | --- | --- |
| *S. aureus* NCTC 8325 | Rhodanese domain-containing sulfurtransferase | YceI-family protein |
| *S. pasteuri* SP1 |  |  |
| *S. warneri* SG1 |  |  |
| *S. epidermidis* E13A |  |  |
| *S. capitis* SK14 |  |  |
| *S. haemolyticus* JCSC1453 |  | hypothetical protein, similar to phosphomannomutase |
| S. lugdunensis HKU09-01 |  | glyoxalase family protein |
| *S. xylosus* C2a | *mvaS*/*mvaC*/*mvaA* | putative secretory antigen |
| *S. saprophyticus* supsp. *saprophyticus* ATCC 15305 |  |  |
| *S. equorum* KS1039 |  | Na+/H+ antiporter NhaC |
| *S. nepalensis* JS1 |  |  |
| S. cohnii SNUDS-2 |  | pseudogene |
| *S. pettenkoferi* FDAARGOS_288 | chromosome partitioning protein, ParB family | nitric oxide dioxygenase |
| *S. sciuri* FDAARGOS_285 | isopentenyl-diphosphate Delta-isomerase | *mvaK1*/*mvaD*/*mvaK2* |
|  |  |  |
